# Physicochemical Properties and Digestion Resistance of Acetylated Starch Obtained from Annealed Starch

**DOI:** 10.3390/polym13234141

**Published:** 2021-11-27

**Authors:** Ewa Zdybel, Aleksandra Wilczak, Małgorzata Kapelko-Żeberska, Ewa Tomaszewska-Ciosk, Artur Gryszkin, Anna Gawrońska, Tomasz Zięba

**Affiliations:** 1 Department of Food Storage and Technology, Faculty of Food Science, Wroclaw University of Environmental and Life Sciences, Chełmońskiego 37, 51-630 Wrocław, Poland; ewa.zdybel@upwr.edu.pl (E.Z.); ewa.tomaszewska-ciosk@upwr.edu.pl (E.T.-C.); artur.gryszkin@upwr.edu.pl (A.G.); tomasz.zieba@upwr.edu.pl (T.Z.); 2 Department of Physico-Chemistry of Microorganisms, Faculty of Biological Sciences, University of Wroclaw, Przybyszewskiego 63-77, 51-148 Wrocław, Poland; aleksandra.wilczak@uwr.edu.pl; 3 Institute of Sport, Tourism and Nutrition, Faculty of Biological Sciences, University of Zielona Góra, Licealna 9, 65-417 Zielona Góra, Poland; agawronska@uz.zgora.pl

**Keywords:** annealing, acetylation, potato starch

## Abstract

One of the examples of physical starch modifications is the retention of a starch suspension in water having a temperature slightly lower than the pasting temperature (annealing). The aim of this study was to investigate the effect of the annealing process performed at various temperatures as the first stage of starch modification. The annealed starch preparations were then esterified using acetic acid anhydride. Finally, the annealed and acetylated starch preparations were determined for their properties. The annealing of starch before acetylation triggered changes in the properties of the modified preparations. It contributed to a higher degree of starch substitution with acetic acid residues and to the increased swelling power of starch. Both these properties were also affected by the annealing temperature. The highest resistance to amylolysis was found in the case of the starch preparation annealed at 53.5 °C and acetylated. The double modification involving annealing and acetylation processes increased the onset and end pasting temperatures compared to the acetylation alone. Similar observations were made for the consistency coefficient and yield point.

## 1. Introduction

Starch is a natural plant raw material applicable in many branches of the industry, which usually makes use of its modified preparations, i.e., starch subjected to various treatments to modify its properties. The methods of starch modification can be divided into physical, chemical, and enzymatic ones [1,2,3,4,5], but also their combinations are employed to achieve desired starch properties [6,7,8]. These modifications allow producing starch preparations with altered properties, including molecular weight, pasting temperature, solubility, viscosity, water binding capability, or resistance to acids and enzymes [9]. One of the examples of physical starch modifications is the retention of a starch suspension in water having a temperature slightly lower than the pasting temperature. This process has been defined as annealing [10].

Its goal is to increase the mobility of molecules; however, without making them turn into the gelatinized form. The annealing process results in the hydration of starch granules, which firstly affects their amorphous and then crystalline regions. Particle mobility increases by analogy, which intensifies the interactions between starch chains and leads to chain ordering. A homogeneous and stable structure of the entire granule is then developed. The swelling of the granule is limited and its form is preserved [10,11,12,13].

The effects of annealing on starch pasting characteristics determined with the DSC are well described in scientific literature [14] and include an increase in transition temperatures, a decrease in a pasting temperature range, and an increase in or no effect on process enthalpy. The annealing contributes to a diminished swelling power of starch granules [15]. In turn, studies have failed to demonstrate its explicit effect on the viscosity of pastes obtained from annealed starch of various botanical origins. The annealing has also been reported to increase the viscosity of pastes made of wheat starch and to decrease that of pastes made of lentil and oat starches [15].

Annealed starch has found multiple applications in the industry [16,17]. Due to its thermal stability and diminished tendency for retrogradation, it is used in food preserves and frozen foods [18]. In turn, Hormdok and Noomhorm [19] described the usability of hydrothermally-treated rice starch for pasta making. It is also worth adding that annealing can contribute to an increased content of resistant starch, especially when coupled with other modifications [20].

The scientific literature indicates the feasibility of performing the annealing process together with other modifications. Zhong et al. [21] investigated effects of rice starch annealing that was heated at a temperature of 45 °C for three days at a starch to water ratio of 1:9, and additionally microwaved. These modifications led to structural and physicochemical changes of starch as well as to increased initial pasting temperature and viscosity of the starch pastes produced. In turn, Iftikhar and Dutta [22] annealed rice starch that had earlier been retrograded; they kept starch in water at 50 °C for 72 h, at a starch to water ratio of 1:6, and by this means achieved new crystalline structures featuring higher thermal stability; whereas Chi et al. [23] modified maize and potato starches by heating dry starches and annealing them at 50 °C for 24 h, at a starch to water ratio of 1:4, and obtained modified preparations with increased resistance to enzymatic activity.

Acetylation is one of the methods of chemical modification that may be coupled with physical processes. It is usually performed using starch acetate (E1420) that is of great importance to the food industry [24] and can be used for a single modification as the so-called acetylated starch (E1420) or in combination with some other chemical modification methods. In turn, acetylated distarch phosphate (E1414), acetylated distarch adipate (E1422), and acetylated oxidized starch (E1451) are applied as food additives [25]. The acetylated starch is produced on the industrial scale by starch esterification in an aqueous suspension with acetic acid anhydride in an alkaline medium. Compared to native starch, it features a lower pasting temperature, pastes made of it are more viscous, and it forms stable gels resistant to retrogradation [26]. The acetylated starch is used as an additive to improve rheological properties of dough. Often, it is also an ingredient of pastas, mayonnaises, ketchups, cottage cheeses, low-fat products, and confectionery products. Oxidized acetylated starch is used to produce jelly candies owing to the clarity of pastes and gels and stability in acidic high-sugar solutions. Acetylated distarch phosphate has been applied in thermally-prepared food products, like soups, ketchups, sauces, cake fillings, or pasteurized cottage cheeses [27]. Acetylation of retrograded starch, precipitated during starch paste freezing and defrosting, is a patented method for resistant starch production [28].

Preparations of acetylated annealed potato starch may be deployed for a slow release of a therapeutic substance [29]. The properties of modified preparations are affected not only by the type but also by the order of performed modifications. Scientific literature lacks reports on the starch subjected to annealing followed by acetylation; hence, the aim of this study was to investigate the effect of the annealing process performed at various temperatures as the first stage of starch modification. The annealed starch preparations were then esterified using acetic acid anhydride. Finally, the annealed and acetylated starch preparations were determined for their properties.

## 2. Materials and Methods

The experimental material included Superior Standard potato starch (PEPEES Łomża S.A., Łomża, Poland) produced in 2020.

Differential scanning calorimetry (DSC) was employed to determine the pasting temperature of potato starch, which reached 62.79 °C.

### 2.1. Annealing

A suspension was prepared from native potato starch, which contained 10 g of starch per 100 g of the solution. The prepared suspension was kept for 48 h at temperatures of 51.0, 53.5, 56.0, 58.5, or 61 °C, under continuous stirring. Afterward, starch was rinsed three times with 5-L portions of distilled water. Starch precipitate was separated from the suspension using a stratos flow-centrifuge (Heraeus Sepatech, Germany), then dried at 30 °C for 24 h, ground, and sieved through a screen with mesh size of 400 μm.

### 2.2. Acetylation

Acetylation was applied to the annealed starch preparations and to native potato starch. Starch dry matter (200 g) and water (1000 g) were placed in a reactor and mixed to achieve a homogenous suspension, the pH value of which was adjusted to pH 8–9 using a 0.5 M NaOH solution. Afterward, 26 mL of acetic acid anhydride were instilled to the starch suspension under continuous stirring. At the same time, the 0.5 M NaOH solution was instilled into the solution to maintain the pH value of the reaction mixture at pH 8–9. Once the whole portion of the anhydride had been added, the mixture was stirred for 15 min and then acidified to pH 5.2–5.6 using a 2.8 M HCl solution. Afterward, the suspension was filtered through a Buchner funnel to enable starch precipitation. The resultant modified starch was rinsed with water until the excess of the reagent had been removed. Then, the preparations were dried at 30 °C for 24 h, ground, and sieved through a screen with mesh size of 400 μm.

### 2.3. Analytical Methods

#### 2.3.1. Determination of the Acetylation Degree of Starch Preparations

Acetylated starch (10 g) was weighed and transferred to a 300 mL conical flask using 65 mL of distilled water. Starch and water were mixed, and then 2–3 drops of phenolphthalein and also 0.1 M NaOH solution were instilled to the mixture until it developed a light pink color which sustained for 1 min. Next, 25 mL of a 0.5 M NaOH solution were added to the mixture, which was then shaken for 35 min. Afterward, the mixture was titrated with a standard HCl solution [30].

The percentage of acetylation A was computed acc. to the following formula:

(1) $$A=\frac{(P_{0}-P_{W})\cdot N_{k}\cdot 0.043\cdot 100}{M_{S}}[\%]$$

where:

*P*_0_—volume of a standard HCl solution used to titrate 25 mL of a 0.5 M NaOH solution [mL].
*P_W_*—volume of a standard HCl solution used to titrate the sample [mL].
*N_k_*—acid titer.
*M_S_*—grams of starch dry matter in the sample.

The degree of acetylation was computed acc. to the following formula:

(2) $$DS=\frac{160\cdot A}{4300-(42\cdot A)}$$

#### 2.3.2. Determination of the Swelling Power of Starch Preparations in Water at a Temperature of 20 °C

The starch preparation (0.3 g) was placed in a 4 mL test tube, to which 2.5 mL of distilled water were added. The content of the flask was conditioned at a temperature of 20 °C for 10 min. Afterward, the samples were centrifuged using an MPW-55 type laboratory centrifuge (MPW Instruments, Warsaw, Poland) at the speed of 5000 rpm and a temperature of 20 °C for 10 min. After centrifugation, the supernatant was collected, and the precipitate left in the test tubes was weighed [29].

Working swelling power was calculated using the following formula:

(3) $$W=\frac{M_{W}-M_{P}}{M_{P}}[\mathrm{g/g}]$$

where:

*M_W_*—weight of the precipitate after centrifugation
*M_P_*—weight of the sample.

#### 2.3.3. Determination of the Resistance of Starch Preparations to the Action of Amyloglucosidase

A 0.72 g/100 g aqueous suspension was prepared from 38 g of starch. It was then heated to the boiling point, cooled, and then the volume of evaporated water was completed. Afterward, 34 mL of an acetate buffer were added, and the sample was placed in a water bath at a temperature of 37 °C. To enable hydrolysis, 4 mL of amyloglucosidase were added; its concentration was adjusted so as to ensure the complete saccharification of gelatinized native potato starch after 120 min of the process. After 120 min, 10 μL of the sample were collected and transferred into a test tube with 1 mL of a reagent for glucose concentration measurement (BioSystem S.A., Barcelona, Spain) containing glucose oxidase and peroxidase. The content of the flask was mixed and incubated at a temperature of 20 °C for 15 min. Then, absorbance was measured at a wavelength of λ = 500 nm using a CECIL CE 2010 colorimeter (Cecil Instruments, England). The measurement results were compared to those achieved for a control sample prepared in an analogous way but without the starch preparation. The absorbance value obtained was compared with the standard curve plotted based on the read outs of absorbance of solutions with a known glucose concentration [31].

Resistance of the starch preparations was computed using the following formula:

(4) $$R=100-\frac{X\cdot 100}{0.4}[g/100\;g]$$

where:

*R*—resistance of starch preparations to amyloglucosidase action (g/100 g).
*X*—content of glucose read out from the standard curve (mg).

#### 2.3.4. Determination of the Characteristics of Phase Transitions of the Starch Preparations Using Differential Scanning Calorimetry (DSC)

Determination was performed using a DSC 822E differential scanning calorimeteter (Mettler Toledo, Germany) [32]. A 10 g starch sample weighed exact to ±0.02 mg was placed on the bottom of a 100 μL aluminum crucible. Then, distilled water was poured into the crucible in the ratio of 3:1 respective to sample weight. The crucible was covered with a lid and conditioned at a temperature of 20 °C for 30 min, then placed in a measuring chamber having a temperature of 20 °C and heated to 100 °C at the heating rate of 4 °C/min. The onset and final temperatures of phase transition (°C) and the heat of this transition (J/g) were determined from thermograms.

#### 2.3.5. Determination of the Flow Curves of Pastes Made of Starch Preparations

Analyses were conducted using an RS 6000 oscillating-rotating viscosimeter Haake (Karlsruhe, Germany) for 5 g/100 g starch suspensions that were heated at 96 °C for 30 min under continuous stirring [33].

Flow curves were plotted for the prepared pastes, at a measurement temperature of 50 °C and a shear rate of 1–300 s^−1^. A hot paste was placed in a system of coaxial cylinders (Z38ALtype) of the RS 100 rheometer, then cooled, and relaxed at the measurement temperature for 15 min. The flow curves plotted were described using the following equations:

Ostwald de Waele:  τ = K ⋯ ẏ^n^ (5)

Casson:   τ^0.5^ = τ _oc_^0.5^ + (ƞ_c_ ⋯ ẏ)^0.5^ (6)

where:

τ—shear stress (Pa)
K—consistency coefficient (Pa·s^n^)
ẏ—shear rate (s^−1^)
n—flow index
τ _oc_—yield point (Pa)
ƞ_c_—Casson’s plastic viscosity (Pa·(s))

### 2.4. Statistical Analysis

Results were statistically analyzed using the Statistica 13.0 PL software package. Based on statistical computations (from at least three parallel replications), values of the least significant differences and standard deviation were calculated, and equations of flow curves were determined. For statistical evaluation, the results were subjected to one-way analysis of variance at a significance level of 0.05. Values of the least significant difference (LSD) between the means were computed using the Duncan’s test at a significance level of 0.05 (StatSoft, Inc., Tulsa, OK, USA, 2011).

## 3. Results and Discussion

**Figure 1:**
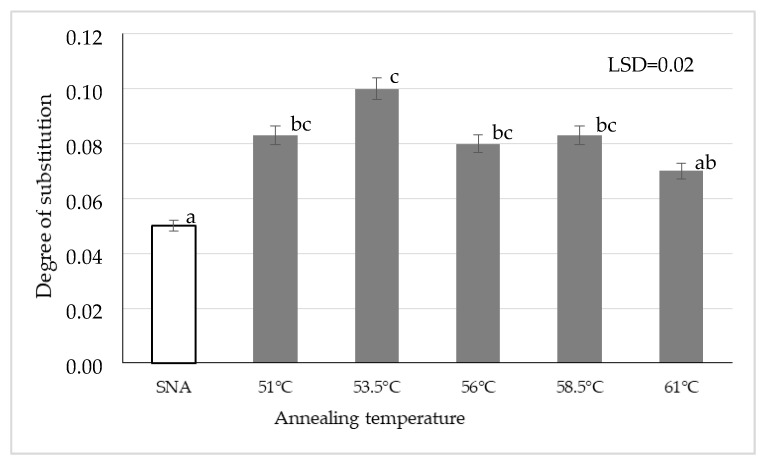
Degree of substitution of acetylated annealed starch preparations depending on the annealing temperature (Different letters mean homogeneous groups at *p* < 0.05).

Figure 1 presents the degree of substitution of starch annealed at various temperatures and then acetylated. Although the annealing process has a significant effect on the possibility of starch substitution with acetic acid residues, there is paucity of literature data regarding the impact of starch annealing on its susceptibility to esterification. The heating of a native starch suspension leads to an increase in the volume of starch granules caused by slow water penetration into their interior. Presumably, the higher substitution degree, compared to the native starch, is due to the loosening of a starch granule structure induced by the breaking of hydrogen bridges accompanied by the release and increasing number of available hydroxyl groups that may be substituted [34]. Among the double-modified preparations, the lowest substitution degree was found for the starch heated at the highest tested temperature (61°), presumably due to starch crystallinity increase during annealing, which reduces starch susceptibility to acetylation [35].

**Figure 2:**
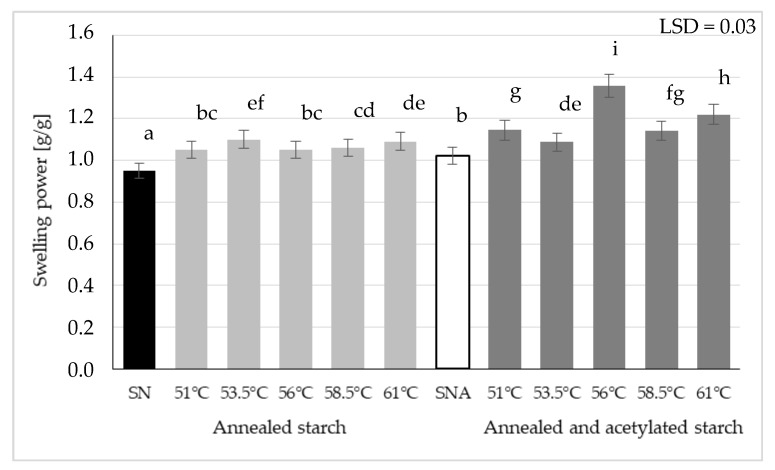
Swelling power of native starch (SN), annealed starch, native acetylated starch (SNA), and annealed and acetylated starch (Different letters mean homogeneous groups at *p* < 0.05).

Figure 2 depicts the effect of acetylation on the swelling power of annealed starch preparations. As a result of starch suspension heating at various temperatures, single-modified starch preparations were obtained having the swelling power of ca. 1 g/g. Their esterification caused an increase in their swelling power, except for the preparation annealed at the temperature of 53.5 °C. According to literature data, esterification increases potato starch affinity to water [36]; however, its impact on the earlier annealed starch has not been fully elucidated. Unpredictable changes that occur during annealing, like re-arrangement of starch granule structure principally in the amorphous region but also the formation of new crystallites or amylose leaching, trigger changes in the properties of annealed starch, which—when subjected to any additional chemical modification—can elicit various effects on the properties of the double-modified starch [37]. These changes can be especially interesting in the case of potato starch, which can develop advanced crystalline structures due to a high number of long chains (B2-B3) [38,39]. Furthermore, as Tomaszewska-Ciosk et al. [40] reports, the dynamics of crystallite formation depends on starch granule size. A broad range of potato starch granule sizes (5–100 µm) may contribute to the uneven increase in the swelling power of its annealed preparations.

The analysis of study results allows concluding that the increased water absorption capacity indicates debilitation of the association potential of polymer chains of starch and increased availability of hydroxyl groups for the formation of new bonds with water molecules. In addition, the micropores occurring naturally on the potato starch surface [41,42], presumably enlarge their sizes, thereby facilitating water penetration into the starch granule interior. However, structural changes evoked in starch granules by starch heating in the excess of water can also lead to both increased availability of hydrophilic groups and the formation of new crystallites, thus diminishing starch capability for water binding [34,36].

Some literature works report various effects of annealing on the swelling power of starches of various botanical origin. Pinto et al. [43] proved that, generally, annealing decreased the swelling power of pinhão starch analyzed in their study; however, this decrease was negligible compared to the non-modified raw material. A decreased swelling power of rice starch was also reported by Dias et al. [44] who annealed it at three different temperatures. In turn, the heating of four cultivars of white yam (a tropical tuber plant) at temperatures below the pasting temperature increased the water absorption capability of one cultivar and decreased it in the case of the three other cultivars studied [45].

**Figure 3:**
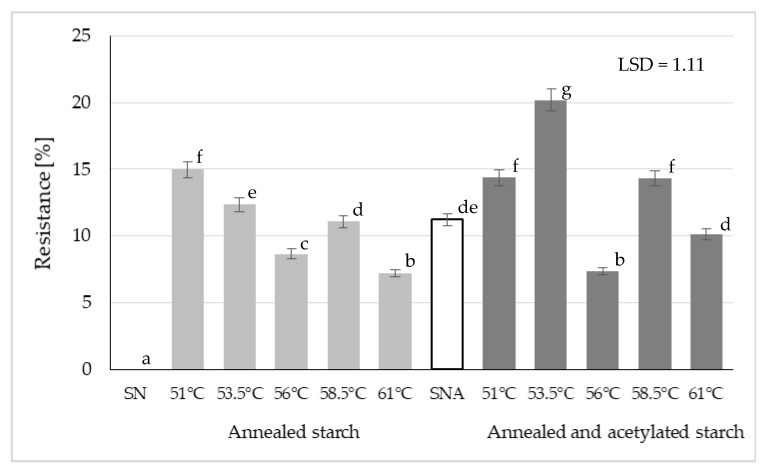
Resistance of native starch (SN), annealed starch, native acetylated starch (SNA), and annealed and acetylated starch (Different letters mean homogeneous groups at *p* < 0.05).

Figure 3 presents the resistance of the analyzed starch preparations to the action of amyloglucosidase. The annealing of native potato starch at various temperatures resulted in the preparations being partly resistant to amyloglucosidase action. Literature works indicate that such hydrothermal modifications as heat moisture treatment (HMT) or annealing increase resistant starch content [29]. The aforementioned authors claim this increase to be due to the rearrangement of starch chains during heating in the excess of water. This type of heating leads to the strengthening of bonds between amylose and amylopectin, thereby increasing starch granule stability, which—in contrast—can inhibit enzymatic hydrolysis [23,46]. Furthermore, annealing can lead to the leaching of amylose chains from amorphous regions of starch granules being especially susceptible to hydrothermal modifications, which probably reinforces the crystalline structure of starch [28]. Ashogbon and Akintayo [37] claimed that the amorphous regions of semicrystalline starch granules represented regions most susceptible to the initial water absorption. Hydration of starch granules leads to their increased mobility in the amorphous regions, while the generated oscillating motion triggers changes in crystallization and interactions between amylose-amylose and amylose-amylopectin chains. With progress in heating/hydration, the initially weaker or imperfect crystallites successively disappear, whereas the other ones become more perfect upon fusion and recrystallization [37]. These are spontaneous, hardly-controllable processes. Many factors contribute to the ultimate effect. Starting from the temperature applied, through the raw material used, to water volume and heating duration.

The resistance of the acetylated potato starch analyzed in the present study reached 11% (Figure 3). This result is consistent with our previous findings [28,47]. The annealing performed before acetylation caused no explicit increase in the resistance of double modified preparations compared to the starch subjected only to chemical modification. However, higher resistance could be noted in the case of the starch preparation annealed at 53.5 °C and acetylated, having a higher degree of substitution at carbon 2 and 3, which makes it difficult for the enzyme to access the hydrolyzed 1–4 glycosidic bond [47]. This shows that acetyl groups block the access of enzymes to the starch chain and that starch resistance increases along with the increasing number of acetyl groups.

Table 1 presents the thermal characteristics of annealed starch as well as acetylated and annealed starch preparations. As reported by other authors [48], starch acetylation decreases phase transition temperatures. This is, most likely, due to the weakening of interactions among starch chains as a result of substitution of hydroxyl groups with acetyl groups that impart amphiphilic character to starch [49], which is in turn associated with the disruption of the crystalline structure of starch during its esterification [26,49]. The acetylation decreased the onset and end pasting temperatures and the phase transition temperature also in the case of the analyzed starch preparations annealed at various temperatures. Even though the crystalline structure of starch had been strengthened by annealing, the successive acetylation of starch weakened its structure, like in the case of native starch.

A Haake oscillating-rotary viscosimeter was used to measure the viscosity of pastes made of modified starch preparations (Table 2). Flow curves were plotted and rheological models were adjusted, which were further used to determine: the consistency coefficient denoting viscosity at the initial shearing phase, paste yield point describing the maximal stress of starch pastes at the null shear rate, and Casson’s plastic viscosity indicating viscosity at the terminal shearing phase [28]. If various starch preparations are to be used in food technology, their rheological properties need to be determined in the first instance, including mainly changes in viscosity of pastes during cooling. This is especially important when starch preparations are applied as thickening agents. The knowledge of factors affecting rheological behavior of starch pastes allows optimizing and standardizing technological processes in the production of foods with starch additives [50,51].

Viscosity measurements demonstrated that the analyzed pastes were characterized by shear-thinned non-Newtonian flow, being typical of starch pastes. Viscosity at the initial shearing phase and yield point of single-modified annealed starch preparations depended on heating temperature. In the study by Wang et al. [52], the annealing of potato starch at a temperature of 50°C caused a viscosity increase, due to greater ordering of starch chains in the amorphous regions of starch. In addition, annealing promotes the swelling of starch granules, from which water is removed together with amylose chains, which may cause an increase in the viscosity of paste made of annealed starch [36].

Another step of modification, i.e., esterification of annealed preparations, contributed to a decrease in the values of coefficients characterizing paste viscosity at the initial shearing phase (except for the preparation annealed at the highest temperature tested), which is due to the diminished structural stability triggered by the introduction of acetyl groups to the starch chain [53,54]. The double modification resulted in lower values of the Casson’s plastic viscosity, indicating greater susceptibility of the double-modified preparations to shear forces compared to the single-modified ones.

## 4. Conclusions

The annealing of starch before acetylation triggered changes in the properties of the modified preparations. It contributed to a higher degree of starch substitution with acetic acid residues and to the increased swelling power of starch. Both these properties were also affected by the annealing temperature. The highest resistance to amylolysis was found for the starch preparation annealed at 53.5 °C and acetylated. The double modification involving annealing and acetylation processes increased the onset and end pasting temperatures compared to the acetylation alone. Similar observations were made for the consistency coefficient and yield point. Preparations of this type may be suitable for the controlled release of bioactive or therapeutic substances.

## Figures and Tables

**Table 1:** Thermal properties of starch preparations annealed at various temperatures and of annealed and acetylated starch preparations.

Preparation Type	Annealing Temperature	Onset Temperature (°C)	End Temperature (°C)	Gelatinization Enthalpy (J/g)
Annealed starch	51 °C	62.66	73.41	14.03
	53.5 °C	65.90	74.66	14.66
	56 °C	66.68	77.81	16.03
	58.5 °C	68.38	76.61	16.06
	61 °C	67.90	77.72	10.48
Acetylated starch	-	48.91	66.02	13.03
Acetylated annealed starch	51 °C	52.36	66.46	11.15
	53.5 °C	54.73	68.37	11.21
	56 °C	61.72	72.42	14.27
	58.5 °C	57.68	70.29	13.81
	61 °C	63.18	76.11	8.62
LSD		0.81	1.47	0.19

**Table 2:** Rheological properties of starch preparations annealed at various temperatures and of annealed and acetylated starch preparations.

Preparation Type	Annealing Temperature	Consistency Coefficient [Pa ⋯ s^n^]	Yield Point [Pa]	Casson’s Plastic Viscosity [Pa ⋯ s]
Annealed starch	51 °C	9.77	25.06	0.42
	53.5 °C	14.00	34.69	0.40
	56 °C	21.78	50.17	0.36
	58.5 °C	20.35	47.20	0.36
	61 °C	9.43	22.64	0.19
Acetylated starch	-	4.78	12.41	0.28
Acetylated annealed starch	51 °C	6.66	17.24	0.30
	53.5 °C	6.45	16.71	0.28
	56 °C	10.67	26.20	0.28
	58.5 °C	8.5	21.96	0.37
	61 °C	13.53	30.78	0.20
LSD		1.09	3.27	0.06

## Data Availability

Data are contained within the article.

## Sample Availability

Samples of the compounds are not available from the authors.
